# How Does Treatment Coverage and Proportion Never Treated Influence the Success of *Schistosoma mansoni* Elimination as a Public Health Problem by 2030?

**DOI:** 10.1093/cid/ciae074

**Published:** 2024-04-25

**Authors:** Klodeta Kura, Nyamai Mutono, Maria-Gloria Basáñez, Benjamin S Collyer, Luc E Coffeng, S M Thumbi, Roy M Anderson

**Affiliations:** London Centre for Neglected Tropical Disease Research, Department of Infectious Disease Epidemiology, School of Public Health, Imperial College London, United Kingdom; Department of Infectious Disease Epidemiology, School of Public Health, Faculty of Medicine, St Mary's Campus, Imperial College London; Medical Research Council Centre for Global Infectious Disease Analysis, School of Public Health, Imperial College London, United Kingdom; Centre for Epidemiological Modelling and Analysis, University of Nairobi, Kenya; Paul G. Allen School for Global Health, Washington State University, Pullman; London Centre for Neglected Tropical Disease Research, Department of Infectious Disease Epidemiology, School of Public Health, Imperial College London, United Kingdom; Department of Infectious Disease Epidemiology, School of Public Health, Faculty of Medicine, St Mary's Campus, Imperial College London; Medical Research Council Centre for Global Infectious Disease Analysis, School of Public Health, Imperial College London, United Kingdom; London Centre for Neglected Tropical Disease Research, Department of Infectious Disease Epidemiology, School of Public Health, Imperial College London, United Kingdom; Department of Infectious Disease Epidemiology, School of Public Health, Faculty of Medicine, St Mary's Campus, Imperial College London; Medical Research Council Centre for Global Infectious Disease Analysis, School of Public Health, Imperial College London, United Kingdom; Department of Public Health, Erasmus University Medical Center, University Medical Center Rotterdam, The Netherlands; Centre for Epidemiological Modelling and Analysis, University of Nairobi, Kenya; Paul G. Allen School for Global Health, Washington State University, Pullman; Institute of Immunology and Infection Research, University of Edinburgh, United Kingdom; London Centre for Neglected Tropical Disease Research, Department of Infectious Disease Epidemiology, School of Public Health, Imperial College London, United Kingdom; Department of Infectious Disease Epidemiology, School of Public Health, Faculty of Medicine, St Mary's Campus, Imperial College London; Medical Research Council Centre for Global Infectious Disease Analysis, School of Public Health, Imperial College London, United Kingdom

**Keywords:** schistosomiasis, mass drug administration, modeling, elimination as a public health problem, never treatment

## Abstract

**Background:**

The 2030 target for schistosomiasis is elimination as a public health problem (EPHP), achieved when the prevalence of heavy-intensity infection among school-aged children (SAC) reduces to <1%. To achieve this, the new World Health Organization guidelines recommend a broader target of population to include pre-SAC and adults. However, the probability of achieving EPHP should be expected to depend on patterns in repeated uptake of mass drug administration by individuals.

**Methods:**

We employed 2 individual-based stochastic models to evaluate the impact of school-based and community-wide treatment and calculated the number of rounds required to achieve EPHP for *Schistosoma mansoni* by considering various levels of the population never treated (NT). We also considered 2 age-intensity profiles, corresponding to a low and high burden of infection in adults.

**Results:**

The number of rounds needed to achieve this target depends on the baseline prevalence and the coverage used. For low- and moderate-transmission areas, EPHP can be achieved within 7 years if NT ≤10% and NT <5%, respectively. In high-transmission areas, community-wide treatment with NT <1% is required to achieve EPHP.

**Conclusions:**

The higher the intensity of transmission, and the lower the treatment coverage, the lower the acceptable value of NT becomes. Using more efficacious treatment regimens would permit NT values to be marginally higher. A balance between target treatment coverage and NT values may be an adequate treatment strategy depending on the epidemiological setting, but striving to increase coverage and/or minimize NT can shorten program duration.

Schistosomiasis is a neglected tropical disease (NTD) caused by the trematode worm *Schistosoma* and transmitted through dermal contact with water contaminated by cercariae, the infectious stage of schistosomes, which are released by the intermediate host snail [1]. The major disease-causing species are *Schistosoma mansoni*, *Schistosoma haematobium*, and *Schistosoma japonicum*. In 2016, schistosomiasis was estimated to account for 1.9 million disability-adjusted life-years, likely a gross underestimate [2, 3]. In 2021, the World Health Organization (WHO) Roadmap on NTDs proposed elimination of schistosomiasis as a public health problem (EPHP; defined as the prevalence of heavy-intensity infection reducing to <1% in school-aged children [SAC]) in all 78 endemic countries by 2030 [4].

Globally, 240 million people reside in areas endemic for schistosomiasis, with 91% of the population at risk living in Africa [5]. Efforts to control and eliminate the disease have been predominantly through preventive chemotherapy treatment with praziquantel (PZQ), which kills the adult worms [4]. Over the years, PZQ has been targeted at SAC (5–14 years of age) in endemic settings, who have the highest risk of infection [6]. To achieve EPHP, the 2022 WHO treatment guidelines recommend inclusion of adults, pre-school-aged children (pre-SAC), and women of reproductive age (including pregnant women from the first trimester), with a target of at least 75% treatment coverage of eligible population per treatment round [7]. The treatment of pre-SAC would require a new, pediatric formulation of PZQ. The proportion of population never treated (NT) after continuous rounds has been reported to influence the success of mass drug administration (MDA) campaigns and the likelihood of achieving elimination targets for helminthiases [8].

Mathematical models have been used to estimate the impact of MDA in achieving disease elimination, while accounting for the precontrol endemicity, treatment coverage, and frequency [9, 10]. However, the implications of the proportion NT are understudied.

In this work, we used mathematical models to provide insights into the impact of NT on achieving the 2030 EPHP target. Specifically, we assessed what proportion of NT would influence the likelihood that schistosomiasis programs achieve EPHP (defined as achieving <1% heavy-intensity prevalence in SAC) target, different treatment regimens (annual, biannual), intensity profile, and coverage levels.

## METHODS

We used 2 individual-based stochastic transmission models developed by Imperial College London (ICL) [11–13] and the University of Oxford (SCHISTOX) [14] to simulate the effect of different levels of NT and MDA coverage among SAC and community on the probability of reaching EPHP for low (<10%), moderate (10%–50%), and high baseline prevalence (>50%) areas as defined by the magnitude of the basic reproduction number, R_0_ (ranging from 1.2 to 4). Both models had similar processes, except for 1 important difference. The ICL model assumed that the number of eggs produced is a nonlinear function (density-dependent egg production) of the female worm burden assuming monogamous sexual reproduction. In contrast, SCHISTOX assumed that the number of eggs produced is proportional to the number of worm pairs (male and female worms). Both models were calibrated with the same baseline settings, by varying the R_0_ in the ICL model, and the overall contact rate (1 term in the denominator of R_0_) in the SCHISTOX model.

We modeled a population of 500 individuals without migration, and various levels of NT (measured after 5 rounds of MDA) among eligible individuals (ranging from 0% to 40%), depending on treatment coverage, following Dyson et al [8].

We assessed the impact of coverage for 60% and 75% of the community (treating those aged ≥2 years) and 75% of SAC (5–14 years), with annual treatment frequency in low- to moderate-prevalence areas and biannual frequency (every 6 months) in high-prevalence areas. We also considered 2 age-intensity profiles of infection, corresponding to low or high burden of infection in adults [10, 15, 16]. Table 1 provides parameter values used in the models.

**Table 1.** Parameter Values for *Schistosoma mansoni*

Parameter	SCHISTOX	ICL	References
Fecundity (eggs/female/sample)	0.34	0.34	[12, 17, 18]
Aggregation parameter	0.04–0.24	0.04–0.24	[6, 16]
Density-dependent fecundity	0.0007	0.0007	[6, 19]
Worm life span, y	5.7	5.7	[6, 12, 20]
Low adult burden setting			
Age-specific contact rates for 0–4, 5–9, 10–15, ≥16 y	0.01, 1.2, 1, 0.02	0.01, 1.2, 1, 0.02	[19, 21]
High adult burden setting			
Age-specific contact rates for 0–4, 5–11, 12–19, ≥20 y	0.01, 0.61, 1, 0.12	0.01, 0.61, 1, 0.12	[19, 21]
Drug efficacy	86.3%	86.3%	[22]
Contact rate	0.03–0.18	…	…
Basic reproduction number	…	1.2–4	…
Population size	500	500	…

Abbreviations: ICL, Imperial College London model; SCHISTOX, University of Oxford model.

At the end of the 20-year treatment duration, we evaluated the heavy-intensity infection to determine whether the proposed EPHP threshold had been met. Each scenario was run 500 times and we considered EPHP to be achieved when 90% of the simulations were below 1% of heavy-intensity prevalence in SAC, which was measured by single Kato-Katz on 2 samples per individual, regardless of the burden of infection in adults.

## RESULTS

**Figure 1.**
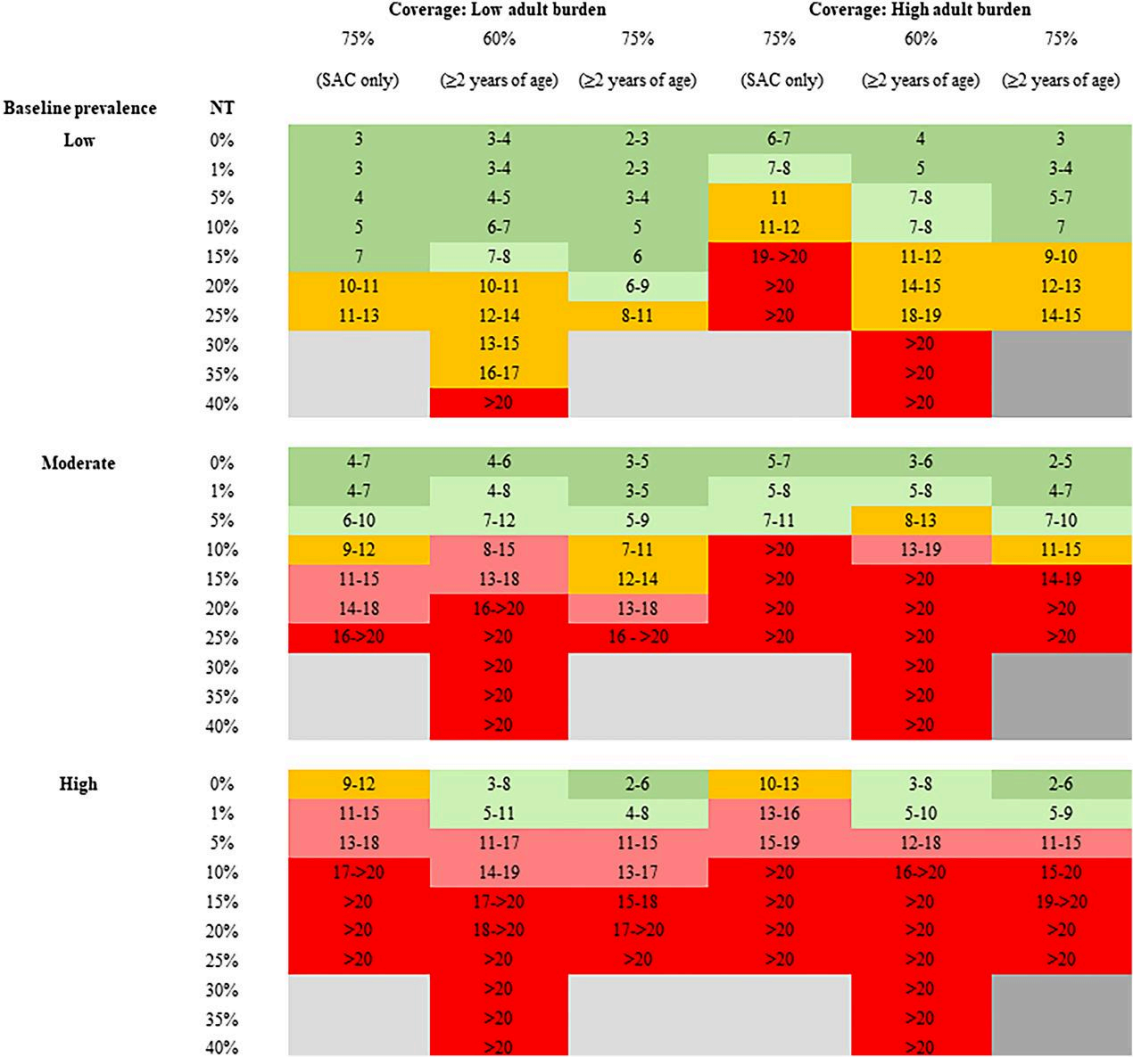
Model-recommended treatment strategies for achieving elimination as a public health problem (EPHP) for low and high burden of infection in adults with different proportions of population never treated (NT). Coverage and NT values are among the eligible population. Results are generated using the Imperial College London and SCHISTOX (University of Oxford) models. Dark green shading shows EPHP achieved within 7 years, orange within 8–14 y, and red within >14 y. Light green shading shows the borderline values within 7 and 8–14 y, and light red shading shows the borderline values within 8–14 y and >14 y. The gray areas show scenarios that cannot be simulated based on the treatment coverage. For low and moderate baseline infection, prevalence treatment frequency is annual; for high baseline infection, prevalence treatment frequency is biannual (and therefore the number of treatment rounds is the number of years multiplied by 2). Low baseline prevalence, <10%; moderate baseline prevalence, 10%–50%; high baseline prevalence, >50%. The number of years is that required to achieve EPHP_90_, defined as 90% of the (500) simulations reaching <1% prevalence of heavy infection intensity (proportion of the population with ≥400 eggs per gram of feces). Abbreviations: NT, never treated; SAC, school-aged children (aged 5–14 y).

In low-prevalence areas, treating 60% of the community with 1% NT would achieve the EPHP target within 5 years, regardless of the burden of infection in adults. Increasing the coverage to 75% of the community increases the probability of elimination (EPHP) and reduces the required number of rounds to achieve the target by 1 year (Figure 1). To achieve EPHP within 7 years, the NT should not exceed 15% in low adult burden settings and 10% in high adult burden settings when treating the community (those aged ≥2 years). Achieving the same target while treating 75% of SAC only would require the NT to be 15% and 1% in settings with low and high burden of infection in adults, respectively.

In moderate-prevalence areas, the EPHP target would be achieved within 7 years for all treatment strategies with NT = 0% (random treatment), regardless of the burden of infection in adults. Increasing the NT to 1% increases the required number of treatment rounds to achieve the target by 1 year, whereas for NT = 10%, >10 years would be required (Figure 1).

In high-prevalence areas, the EPHP target would be achieved within 7 years by treating 75% of the community (those aged ≥2 years), regardless of the burden of infection in adults provided that NT = 0%. Treating SAC only in high-prevalence areas would not achieve EPHP target within 7 years, and a proportion NT = 1% would require >12 years of biannual treatment to achieve EPHP.

For a low burden of infection in adults, the success of a SAC-only treatment program depends on the baseline prevalence and the NT proportion. For baseline prevalence above a threshold (67% for ICL and 76% for SCHISTOX), an increase in SAC coverage and inclusion of adults is recommended to achieve the target within 7 years of treatment. Specific results from each model are presented in Supplementary Table 1. Elimination probability (EPHP) results for a high-prevalence setting when NT = 0% are shown in Supplementary Figure 1.

## DISCUSSION

We find that community-wide treatment including the use of the new formulation of PZQ to treat pre-SAC can achieve elimination as a public health problem within a short time frame provided MDA coverage is good and individual compliance to treatment is effectively random at each round. Independent of MDA coverage, the outcome depends on the burden of infection in adults and the baseline prevalence (determined by the magnitude of R_0_). The higher the MDA coverage and treatment compliance (Figure 1), the lower the number of rounds required to achieve this target.

Despite the target being achieved in some areas for different treatment strategies, there is a high risk of resurgence following MDA cessation if control efforts are not maintained. The worm aggregation in a community is unevenly distributed, and it is challenging to measure the variability after MDA treatment. The worm aggregation may increase after many rounds of MDA if there is a small proportion of people with heavy-intensity infection that has never been treated. These individuals are a reservoir of infection and increase the risk of resurgence. To prevent resurgence, it is important to maintain EPHP with reduced efforts (less frequent or lower coverage of MDA) or move towards the interruption of transmission goal [10, 21]. The likelihood of maintaining the EPHP target will critically depend on the strategy adopted and the transmission setting, whereby more intense efforts are required in high-transmission areas.

For a given NT value, treatment coverage is an important driver of program duration: the greater the coverage of eligible population, the shorter the projected number of years to achieve EPHP. This is because as prevalence falls in the majority of the population, infection levels in NT individuals also decrease due to a lower incidence of new infections through lower transmission, and natural death of existing worms that are replenished at a lower rate. As long as there are only a few NT individuals harboring reproductively active worms, transmission in the overall population may fall sufficiently low that eventually, infection levels in NT individuals are not able to sustain infection for the entire population above 1% prevalence of heavy-intensity infection in SAC.

There is a clear need for more studies of individual compliance patterns in PZQ MDA-treated communities, as very few longitudinal studies of compliance have been conducted [23]. In future work we will use data from the ongoing Geshiyaro project in Ethiopia, which is following a large population treated with PZQ over many rounds of MDA and recording individual adherence behaviors [24].

While our models consider closed populations, human movement between communities (either as short-term commuting or long-term migration, including population displacement as a result of civil unrest and/or climate change) can hamper the success of MDA programs by reducing the probability of elimination (or increase the rate of resurgence upon cessation of MDA) due to spatial diffusion between communities with differing levels of treatment coverage [22]. This is particularly important when programs transition from EPHP towards elimination of transmission. It is also important to consider the sensitivity of the diagnostic technique. In this study, the prevalence of infection was measured by Kato-Katz, which has a low sensitivity in detecting infection at very low prevalence areas. Alternative diagnostic techniques such as the point-of-care circulating cathodic antigen could be helpful as it has a greater sensitivity at low prevalence than Kato-Katz [25–27].

We acknowledge limited PZQ supplies (which are donated), and therefore considering a community-wide treatment or high coverage levels may not be feasible in all settings. Additional interventions, such as improving water, sanitation, and hygiene (WASH), the future use of an efficacious vaccine (if one were to become available), and/or snail control could reduce the number of years of MDA required to achieve EPHP. The schistosomiasis control and elimination program in China can provide valuable insights that can be applied in other countries affected by schistosomiasis.

The ICL model was also used to produce results for *S. haematobium* infection (Supplementary Table 2 and Supplementary Figure 2), which are similar to the *S. mansoni* results with low adult burden.

## Supplementary Data

Supplementary materials are available at *Clinical Infectious Diseases* online. Consisting of data provided by the authors to benefit the reader, the posted materials are not copyedited and are the sole responsibility of the authors, so questions or comments should be addressed to the corresponding author.

## Supplementary Material

ciae074_Supplementary_Data

## Notes

***Author contributions.*** K. K.: conceptualization, formal analysis, investigation, methodology, visualization, project administration, writing—original draft, and writing—review and editing. N. M.: conceptualization, formal analysis, investigation, methodology, visualization, writing—original draft, and writing—review and editing. B. S. C: formal analysis. M.-G. B: conceptualization and writing—review and editing. L. E. C.: conceptualization, methodology, writing—original draft, and writing—review and editing. S. M. T. and R. M. A.: conceptualization, methodology, visualization, supervision, and writing—review and editing.

***Financial support.*** This work was supported by the Bill & Melinda Gates Foundation through the NTD Modelling Consortium (grant number INV-030046). K. K., M.-G. B., and R. M. A. also acknowledge funding from the MRC Centre for Global Infectious Disease Analysis (MR/X020258/1), funded by the UK Medical Research Council (MRC). This UK-funded award is carried out in the frame of the Global Health EDCTP3 Joint Undertaking.

***Supplement sponsorship.*** This article appears as part of the supplement “New Tools and Nuanced Interventions to Accelerate Achievement of 2030 Roadmap for Neglected Tropical Diseases,” sponsored by funding of Professor T. Déirdre Hollingsworth's research by the Li Ka Shing Foundation at the Big Data Institute, Li Ka Shing Centre for Health Information and Discovery, University of Oxford; and funding of the NTD Modelling Consortium by the Bill & Melinda Gates Foundation (INV-030046).
