# Supplementary material for: How Does Treatment Coverage and Proportion Never Treated Influence the Success of Schistosoma mansoni Elimination as a Public Health Problem by 2030?
Source: Clin Infect Dis. 2024 Apr 25;78(Suppl 2):S126–30. doi: 10.1093/cid/ciae074 (PMC11045018; doi:10.1093/cid/ciae074)
Supplement: ciae074_Supplementary_Data [file ciae074_supplementary_data.docx]

**Supplementary appendix**

Supplement to:

**How does treatment coverage and proportion never treated influence the success of *Schistosoma mansoni* elimination as a public health problem by 2030?**

Klodeta Kura^1,2,3*^, Nyamai Mutono^4,5*^, Maria-Gloria Basáñez^1,2,3^, Benjamin S. Collyer^1,2,3^, Luc E. Coffeng^6^, S.M. Thumbi^4,5,7^ and Roy M. Anderson^1,2,3^

^1^ London Centre for Neglected Tropical Disease Research, London, UK

^2^ Department of Infectious Disease Epidemiology, School of Public Health, Faculty of Medicine, St Mary’s Campus, Imperial College London, UK

^3^ MRC Centre for Global Infectious Disease Analysis, School of Public Health, Imperial College London, London, UK

^4^ Centre for Epidemiological Modelling and Analysis, University of Nairobi, Nairobi, Kenya

^5^ Paul G Allen School for Global Health, Washington State University, Pullman, USA

^6^ Department of Public Health, Erasmus MC, University Medical Center Rotterdam, Rotterdam, The Netherlands

^7^ Institute of Immunology and Infection Research, University of Edinburgh, Edinburgh, UK

^*^Co-first authors

Correspondence to: k.kura@imperial.ac.uk; mutono.nyamai@uonbi.ac.ke

**Table S1**: Model recommended treatment strategies for achieving elimination as a public health problem for low and high adult burden of infection with different proportions of the eligible population never treated. Results are generated using the ICL(second row) and SCHISTOX (first row) models.

|  |  | **Coverage** | | |
| --- | --- | --- | --- | --- |
|  |  | **75% SAC** | **60% Community** | **75% Community** |
| Baseline prevalence | NT |  |  |  |
| Low  (8%) | 0% | SCHISTOX: 3 years  ICL: 3 years | SCHISTOX: 4 years  ICL: 3 years | SCHISTOX: 3 years  ICL:2 years |
|  | 1% | 3  3 | 4  3 | 3  2 |
|  | 5% | 4  4 | 5  4 | 4  3 |
|  | 10% | 5  5 | 7  6 | 5  5 |
|  | 15% | 7  7 | 8  7 | 6  6 |
|  | 20% | 10  11 | 10  11 | 6  9 |
|  | 25% | 11  13 | 12  14 | 8  11 |
|  | 30% |  | 13  15 |  |
|  | 35% |  | 16  17 |  |
|  | 40% |  | >20  >20 |  |
| Moderate  (13%-49%) | 0% | 5-7  4-5 | 5-6  4-5 | 4-5  3 |
|  | 1% | 5-7  4-5 | 5-8  4-7 | 4-5  3-4 |
|  | 5% | 7-10  6-9 | 9-12  7-10 | 6-9  5-7 |
|  | 10% | 10-13  9-12 | 12-15  8-12 | 9-11  7-11 |
|  | 15% | 12-15  11-14 | 14-18  13-17 | 12-14  12-13 |
|  | 20% | 14-18  15-18 | 16- >20  16->20 | 13-17  14-18 |
|  | 25% | 16->20  17->20 | >20  >20 | 16- >20  16->20 |
|  | 30% |  | >20  >20 |  |
|  | 35% |  | >20  >20 |  |
|  | 40% |  | >20  >20 |  |
| High  (51%-70%) | 0% | 9-12  9-12 | 5-7  3-8 | 4-6  2-5 |
|  | 1% | 11-14  12-15 | 7-11  5-11 | 6-8  4-7 |
|  | 5% | 13-15  14-18 | 11-15  12-17 | 11-15  11-14 |
|  | 10% | 17->20  18->20 | 14-19  15-20 | 13-17  13-17 |
|  | 15% | >20  >20 | 17->20  17->20 | 15-17  15-18 |
|  | 20% | >20  >20 | 18->20  18->20 | 17->20  17->20 |
|  | 25% | >20  >20 | >20  >20 | >20  >20 |
|  | 30% |  | >20  >20 |  |
|  | 35% |  | >20  >20 |  |
|  | 40% |  | >20  >20 |  |

**Table S2.** Parameter values for ICL *Schistosoma haematobium* model

| **Parameter** | **Value** | **Reference** |
| --- | --- | --- |
| Fecundity (eggs/female/10 ml sample) | 3.6 | [1], [2] |
| Aggregation parameter | 0.04-0.24 | [3] |
| Density dependent fecundity | 0.0006 | [4] |
| Worm life span (years) | 4 | [5], [6] |
| Age specific contact rates for 0-5, 5-10, 10+ years old | 0.3, 1, 0.02 | [7] |
| Drug efficacy | 94% | [8] |
| Aggregation of diagnostic | 0.5 | [3] |
| Basic reproduction number | 1.2-2 | - |
| Population size | 500 | - |


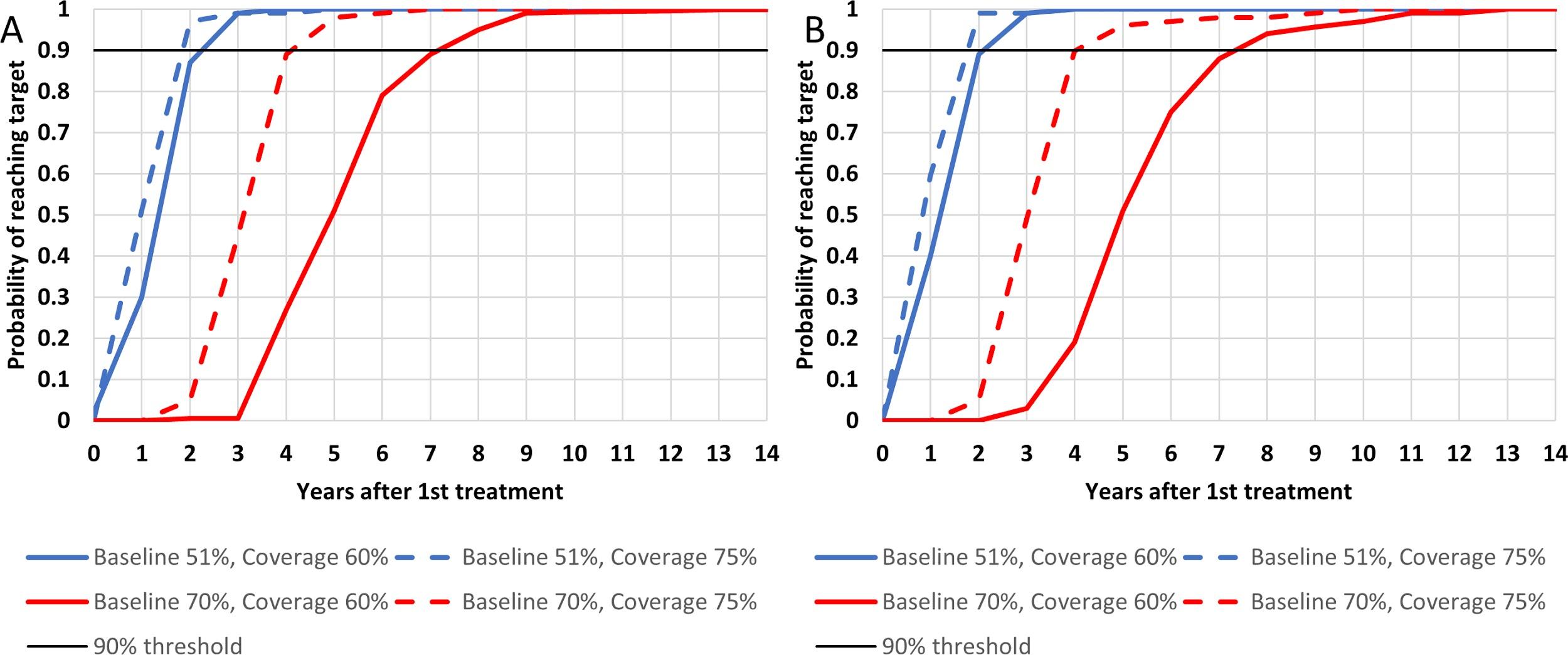


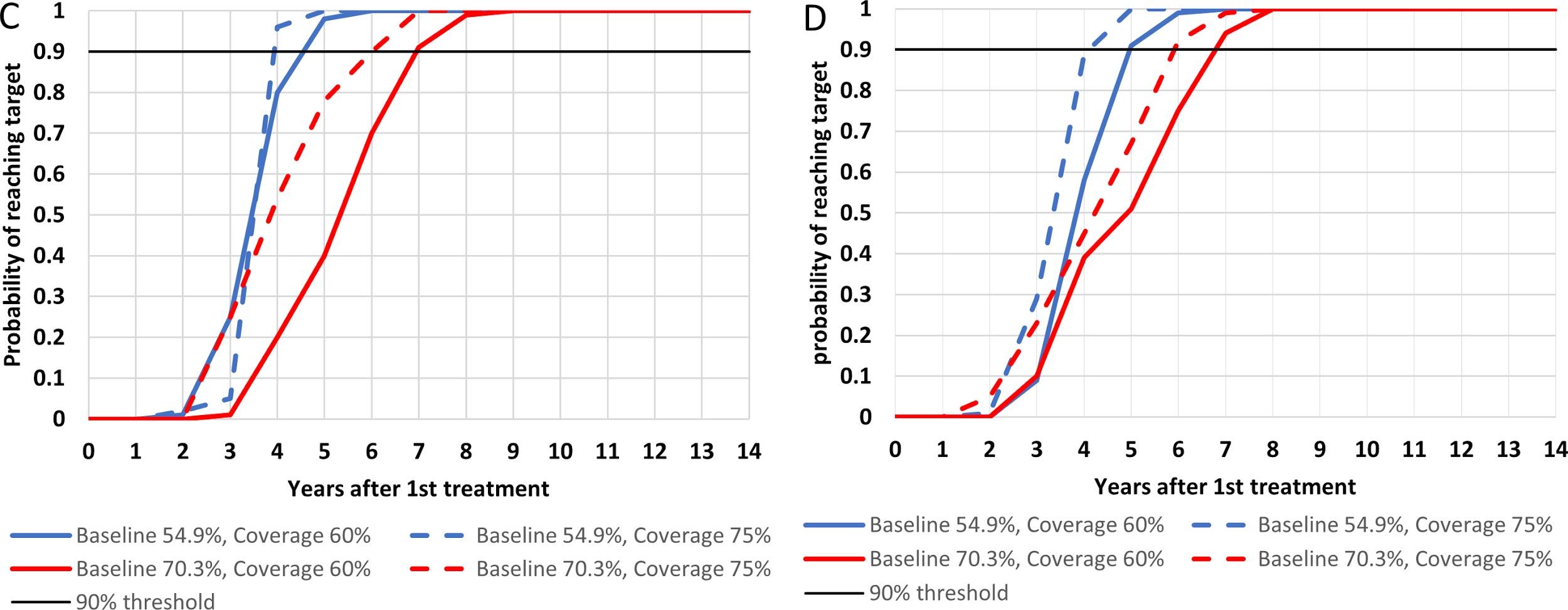


**Figure S1:** The probability of elimination (proportion of 500 model runs that achieve EPHP after each treatment round) for a high prevalence area with 60% community-wide treatment (solid line) or 75% community-wide treatment (dashed line) assuming: (A, C) low adult burden of infection; (B, D) high adult burden of infection when NT=0. Results shown are generated by the ICL model (top row) and the SCHISTOX model (bottom row).

**Figure S2:** *Schistosoma haematobium* results. Model recommended treatment strategies for achieving elimination of *Schistosoma haematobium* infection as a public health problem (EPHP) for low and high burden of infection in adults with different proportions of population never treated (NT). Coverage and NT values are among eligible population. Results are generated using the ICL model. The dark green shade shows EPHP achieved within seven years, orange within eight to 14 years, and red above 14 years. The grey areas show scenarios that cannot be simulated, based on the treatment coverage.

SAC: School aged children, 5 – 14 years; for low and moderate baseline infection prevalence treatment frequency is annual; for high baseline infection prevalence treatment frequency is biannual (and therefore the number of treatment rounds is the number of years multiplied by two). Low baseline prevalence = <10%; moderate baseline prevalence = 10–50%; high baseline prevalence > 50%. The number of years is that required to achieve EPHP_90_, defined as 90% of the (500) simulations reaching <1% prevalence of heavy infection intensity (proportion of the population with ≥50 eggs per 10ml urine sample).

**References**

[1] A. W. Cheever, “A quantitative post-mortem study of Schistosomiasis mansoni in man.,” *American Journal of Tropical Medicine and Hygiene*, vol. 17, no. 1, pp. 38–64, 1968.

[2] M. D. French *et al.*, “Estimation of changes in the force of infection for intestinal and urogenital schistosomiasis in countries with schistosomiasis control initiative-assisted programmes,” *Parasit Vectors*, vol. 8, no. 1, p. 558, 2015, doi: 10.1186/s13071-015-1138-1.

[3] J. E. Truscott *et al.*, “A comparison of two mathematical models of the impact of mass drug administration on the transmission and control of schistosomiasis,” *Epidemics*, vol. 18, pp. 29–37, 2017, doi: https://doi.org/10.1016/j.epidem.2017.02.003.

[4] R. M. Anderson, H. C. Turner, S. H. Farrell, and J. E. Truscott, “Studies of the Transmission Dynamics, Mathematical Model Development and the Control of Schistosome Parasites by Mass Drug Administration in Human Communities,” *Adv Parasitol*, vol. 94, pp. 199–246, Jan. 2016, doi: 10.1016/bs.apar.2016.06.003.

[5] R. M. Anderson and R. M. May, “Helminth Infections of Humans: Mathematical Models, Population Dynamics, and Control,” *Adv Parasitol*, vol. 24, no. C, 1985, doi: 10.1016/S0065-308X(08)60561-8.

[6] R. M. Anderson and R. M. May, *Infectious diseases of humans: dynamics and control*. Oxford University Press, 1992. [Online]. Available: https://global.oup.com/academic/product/infectious-diseases-of-humans-9780198540403?cc=gb&lang=en&

[7] K. Kura, R. J. Hardwick, J. E. Truscott, J. Toor, T. D. Hollingsworth, and R. M. Anderson, “The impact of mass drug administration on Schistosoma haematobium infection: what is required to achieve morbidity control and elimination?,” *Parasit Vectors*, vol. 13, no. 1, p. 554, 2020, doi: 10.1186/s13071-020-04409-3.

[8] J. Zwang and P. L. Olliaro, “Clinical Efficacy and Tolerability of Praziquantel for Intestinal and Urinary Schistosomiasis—A Meta-analysis of Comparative and Non-comparative Clinical Trials,” *PLoS Negl Trop Dis*, vol. 8, no. 11, pp. e3286-, Nov. 2014, [Online]. Available: https://doi.org/10.1371/journal.pntd.0003286
